# ‘Everyone has a secret they keep close to their hearts’: challenges faced by adolescents living with HIV infection at the Kenyan coast

**DOI:** 10.1186/s12889-016-2854-y

**Published:** 2016-02-29

**Authors:** Amina Abubakar, Fons J. R. Van de Vijver, Ronald Fischer, Amin S. Hassan, Joseph K Gona, Judith Tumaini Dzombo, Grace Bomu, Khamis Katana, Charles R. Newton

**Affiliations:** Centre for Geographic Medicine (Coast), Kenya Medical Research Institute, Kilifi, Kenya; Tilburg University, Tilburg, Netherlands; Lancaster University, Lancaster, UK; North-West University, Potchefstroom, South Africa; University of Queensland, Brisbane, Australia; Victoria University of Wellington, Wellington, New Zealand; University of Oxford, Oxford, UK; Aarhus University, Aarhus, Denmark

**Keywords:** HIV, Adolescents, In-depth interviews, Kenya

## Abstract

**Background:**

The upsurge in the uptake of antiretroviral therapy (ART) has led to a significant increase in the survival of vertically acquired HIV infected children, many of whom are currently living into adolescence and early adulthood. However little if anything is known of the lived experiences and the challenges faced by HIV positive adolescents in the African context. We set out to investigate psychosocial challenges faced by HIV infected adolescents on the Kenyan coast.

**Methods:**

A total of 44 participants (12 HIV-infected adolescents, 7 HIV uninfected adolescents, and 25 key informants) took part in this qualitative study, using individually administered in-depth interviews. A framework approach was used to analyze the data using NVIVO software.

**Results:**

We observed that the challenges faced by adolescents in rural Kenya could be placed into six major themes: poverty, poor mental and physical health, the lack of a school system that is responsive to their needs, challenges in how to disclose to peers and family members, high levels of stigma in its various forms, and challenges of medical adherence leading to the need for close monitoring.

**Conclusion:**

In this African community, vertically acquired HIV-infected adolescents face a complex set of social, economic and medical challenges. Our study points to the urgent need to develop multisectorial intervention support programmes to fully address these challenges.

**Electronic supplementary material:**

The online version of this article (doi:10.1186/s12889-016-2854-y) contains supplementary material, which is available to authorized users.

## Background

By the end of 2012, an estimated 2.3 million children were living with HIV infection worldwide; of whom, 90 % live in sub-Saharan Africa [1]. The upsurge in the uptake of antiretroviral therapy (ART) has led to a significant increase in the survival of vertically acquired HIV infected children, many of whom are currently living into adolescence and early adulthood [2]. The dramatic increase in life expectancy in this population has led to a rise in interest in understanding the psychosocial sequelae of HIV infection in this age group, such as mental health [3, 4], cognitive development [5], sexuality [6], reproductive health [7], day-to-day challenges [8], life skills [9], and barriers to their care [10]. Despite the increased interest in understanding these challenges, the day-to-day experiences of African adolescents living with HIV infection remain largely understudied.

Experiences of HIV infected adolescents within the school system have largely been under-investigated in the African context. Understanding the educational experiences of HIV infected adolescents is important since schools constitute an important developmental context for this age group [11]. Children between the ages of 6-18 years spend most of their days in school; hence positively influencing the quality of time in school could greatly increase their subjective well-being. Additionally, schools help to shape cognitive, social, emotional, and behavioral functioning of these children [11]. For adolescents living with HIV, school conditions may facilitate or hinder adherence to ART and other interventions. For instance, a recent study from Rwanda noted that attending a boarding school (where almost 45 % of HIV infected adolescents in their sample were studying) was one of the major barriers to medical adherence [10].

Given these knowledge gaps, we designed a qualitative study to investigate the experiences and challenges of HIV infected adolescents at the Kenyan coast. Specifically, we set out to answer the following research question: What are the psychosocial challenges faced by HIV infected adolescents?

## Methods

### Study design

The study was undertaken at the Centre for Geographic Medicine Research-Coast in Kilifi, Kenya. A cross-sectional qualitative study design was used. HIV-infected adolescents and their caregivers were recruited from the HIV clinics at the Kilifi County Hospital, Vipingo Health Centre, and Bamba Health Centre. HIV-uninfected adolescents were randomly selected from the community until we reached our target number. Some of the HIV-uninfected adolescents knew someone living with HIV. Key informants included health service providers and community health workers from the participating hospitals, and teachers and education administrators from local secondary schools from within Kilifi County, Kenya.

### Data collection

Semi-structured key informant interviews were used to collect the data. A checklist of questions was developed by the research team through discussion and consensus. The key questions were “what are the challenges experienced by adolescents living with HIV’ “How has HIV impacted on the lives of the adolescents’ and ‘how have they dealt with the knowledge that they are living with HIV’ (See Additional file 1 for a more complete list of the questions). The questions were phrased to suit the type of respondent we were talking to. These questions were clarified and probes included as deemed necessary. Each interview took approximately one hour. The interviews were largely conducted by one of the co-authors, GB assisted JG. The interviews were conducted in the language that the interviewee was most comfortable with, which was either English or Kiswahili.

### Data management and analysis

The final transcripts used for analysis were based on the audio-taped materials and supplemented with written notes. Data was analyzed with the assistance of NVIVO 10 software programme according to the framework analysis [12, 13]. The transcripts of the interviews were reviewed and read during which a coding scheme was developed. The first author (AA) developed coding schemes and identified themes. The themes identified were then evaluated, checked, and discussed by one of the authors (JG), who also independently coded five randomly selected transcripts.

### Ethics statement

The Kenya Medical Research Institute National Scientific and Ethical Committees approved the study (SSC no.2011). Written informed consent was obtained from key informants prior to participation. Adolescents provided written informed assent while their caregivers provided written informed consent.

## Results

### Study population

Twelve HIV infected adolescents (3 females) aged 12–17 years (mean age = 14.50, SD = 1.78) and 7 HIV uninfected adolescent (5 females) aged 12–17 years (mean age = 15.00, SD = 2.23) took part in this study. Key informants included 11 caregivers of HIV infected adolescents (2 fathers, 4 grandmothers and 5 mothers), health service providers and community health workers (*n* = 8), teachers and education administrators (*n* = 6) all from within Kilifi County.

### Challenges faced by HIV infected adolescents

The following major themes emerged in our discussions with HIV infected and uninfected adolescents and key informants: poverty, poor mental and physical health, poor handling of disclosure, high levels of stigma in its various forms, lack of close clinical follow-up and monitoring to ensure medical adherence, and lack of a school system that is responsive to their needs. Below we discuss each of these challenges.

### Poverty as a salient challenge for families with HIV

Many of the HIV infected adolescents mentioned poverty related issues, such as lack of proper meals, clothing, and school fees as a major challenge.

*“I lack school fees, food and fare…… to come here [to the health clinic]” (HIV infected adolescent, Female, 13 years)*

*“For instance, now there is problem with (access to) food; sometimes he takes medication only and goes to school as he has nothing to eat and at lunch time when he comes he takes porridge and goes back to school. He does not take nutritious foods but he is being helped by God.” [Grandmother, caregiver of an HIV infected adolescent]*

*“The main challenge, they are complaining a lot about hunger. They say because of medication they need a lot of food and you see most of their guardians are not financially able…….’ [Community health worker, Female]*

### Poor mental and physical health

Although HIV infected adolescents themselves rarely mentioned facing problems with physical and mental health, these were raised frequently by their service providers and caregivers both at hospital and at home:

*“Ok we know HIV affects child growth and development. This is due to recurrent infections they get during the course of illness. So you find most of them have, say, things like skin rash and maybe delayed milestones……” [Medical officer, Male]*

*“They are generally scared, they are anxious…” [Hospital based health worker, Female].*

*“He is always lonely and unhappy until sometimes I cheat him [I tell him] that do not worry you no longer have the virus …” [Grandmother, caregiver of an HIV infected adolescent]*

### Confronting a school system that is not responsive to their needs

HIV infected adolescents have to deal with a school system that is not very responsive to their specific medical, developmental and educational needs. At the very least adolescents had to miss one day of schooling every month to attend the clinic:

*“Yes he attends school regularly, except for the clinic days…” [Father, carer of an HIV infected adolescent].*

More serious challenges included, among other things, lagging behind in education, discrimination and isolation at school (this will be discussed more in the section on stigma), hiding to take medication, and teachers breaking confidentiality when they become aware of the student’s HIV status.

*“He used to go to school, but does not learn anything ………at the end of the year he is retained in the same class…” [Community based health worker, Female].*

*“… when they are at school, we have been informed that some of them have to hide and take their medicines in the toilet.…..” [Hospital based health worker, Female].*

Of the 12 HIV infected adolescents interviewed, 2 (15 %) were not enrolled in school, mainly because of HIV related illness. Continued ill-health had kept one of the adolescents out of school for more than 3 years, but s/he was still hopeful of going back to school. Another adolescent had clinically stabilized following initiation of ART; however, going back to school meant joining a lower class and being in class with much younger children, which was socially awkward. Key informants especially community health workers noted this challenge independently. They informed us that HIV infected adolescents felt discouraged when they could not continue with their education following prolonged periods of severe ill-health.

*“Because of ill-health…. just like I told you before when he wanted to go back to school he was told to go back to class 2, he refused………… he refused because they [class 2 pupils] are very young and he is older” [Grandmother, caregiver of an HIV infected adolescent]*

*“….in my youth, for me to miss an education, makes my heart feel a lot of pain [is a painful experience]” [HIV infected adolescent, Male, 17 years].*

### Partial disclosure to family and peers preferred to avoid stigma, yet it contributed to anxiety and complicated social relationships

As part of the inclusion criteria for the current study, all adolescents had to be fully aware of their HIV status. We were interested in their reaction when they *first learnt* that they were HIV infected, how many people in their immediate environment were aware of their HIV status and factors that influenced their decision to disclose or not to disclose their HIV status to those in their immediate environment. In general the adolescents reported that they handled learning of their status with acceptance.

*“I just took it easy.” [HIV infected adolescent, Male 12 years old]*

However there were a few who reported experiencing distress such as crying or contemplating suicide. Having a support system, such as a parent whom they could share with or a counselor, helped them get over their distress fairly quickly.

*“I was sad….. I cried…… but after that [a counseling session], I felt encouraged by my doctor and grandmother [HIV infected adolescent, Male, 13 years old]*

*Respondent “I thought of going to hang myself…” (HIV infected adolescent, Female, 13 years old)*

*Interviewer: So what made you change your mind about hanging yourself?*

*Respondent: I talked to my mother …..*

However, disclosing their HIV status to people within their immediate environment seemed to be a challenge for these adolescents. Their HIV status was seen as a secret that needed to be guarded. Partial disclosure (where only a few people who must know are informed about the HIV status) is the preferred strategy for most of our HIV infected participants:

*“And I won’t tell them, everyone has a secret that they keep close to their heart” (HIV infected adolescent, Male, 16 years old)*

Many adolescents and their caregivers believed that if they mentioned their status to peers and teachers, they risked rejection, isolation, and stigmatization. Both the adolescents and their caregivers perceived not disclosing their HIV status to others as the best way to avoid negative social consequences:

*“They [teachers, peers] may isolate, discriminate against me” (HIV infected adolescent)*

*“I think that if I tell other children, they might end up treating him badly or have negative attitudes towards him”. (Caregiver, HIV infected adolescent)*

*“…You know for kids they might be playing and then they start rebuking her/him that s/he has HIV and AIDS.” (Caregiver HIV infected adolescents)*

The potential problems that may arise when not disclosing one’s status in their immediate environment came up during our discussions. Some of the problems included constant worrying about their secret being revealed and managing medication in secrecy.

*“I am worried because we are two, we were four siblings, but two have died. I look at my younger brother and my heart is filled with lots of questions, now my brother does not know [of the participant’s HIV status], what will happen when he knows?” (HIV infected adolescent, Male, 17 years)*

*“ … He decided to get the children from [region a] where they were receiving medication and brought them to [region b] to attend school but did not tell the new wife that the kids were on ART, or even their HIV status… now imagine a young kid knows nothing and being told to not take his drugs by the father.” (Hospital based health worker, Female).*

### Stigma presented itself in many different covert patterns

**Fig. 1:**
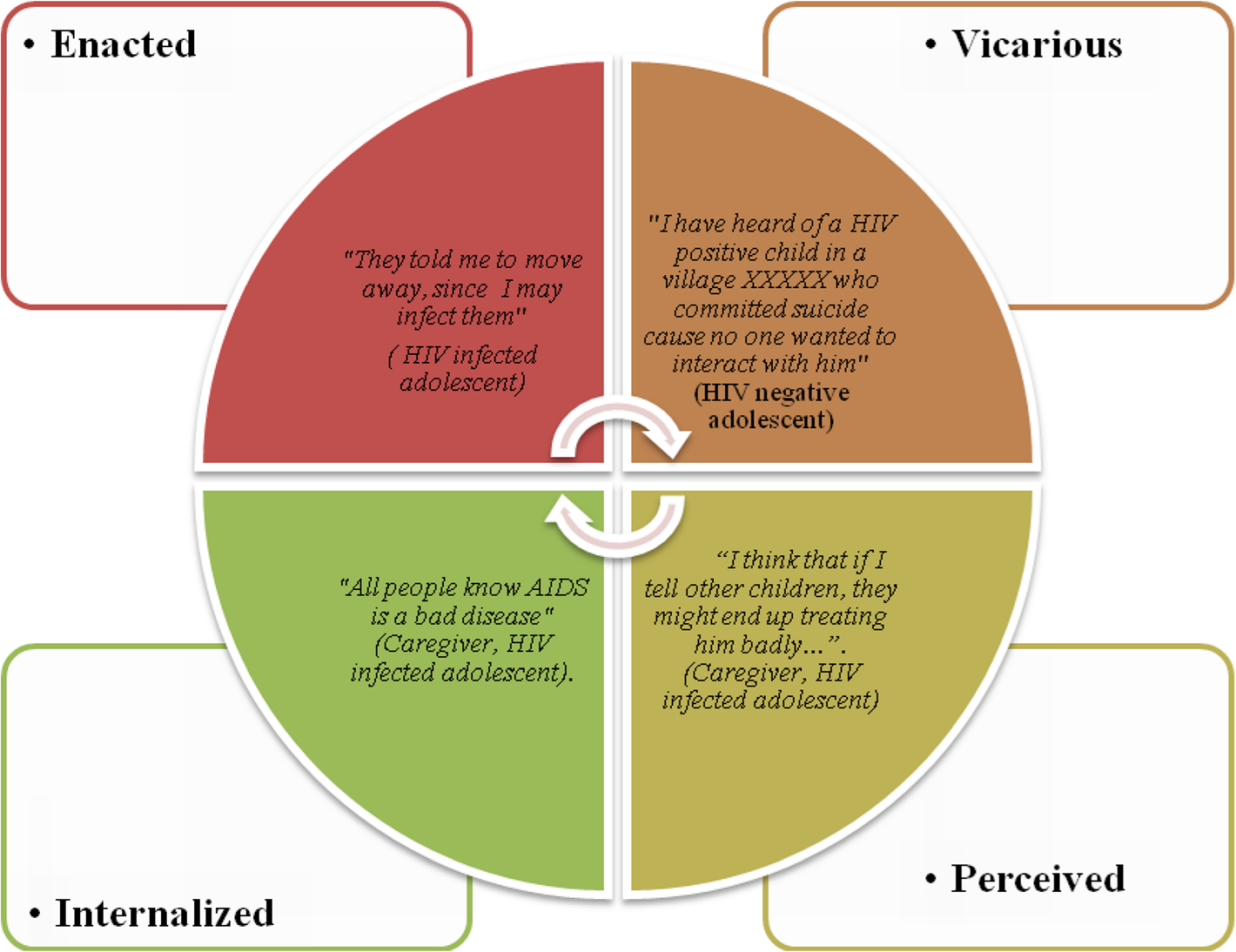
Various forms of stigma arising in our dataset

From our interviews, it was clear that the overt forms of stigma are less common due to raised awareness. However, covert forms appeared to be prevalent. With a social-cognitive perspective, the forms of stigma observed can be categorized into four types (Fig. 1).

For instance**,** an analysis of the discourse on HIV by some of the participants (such as educationalists, HIV uninfected adolescents, and HIV infected adolescents, and their caregiver) illustrated that they may potentially hold views, opinions’ or beliefs that are stigmatizing. As one educationalist said that most HIV infected adolescents contracted the disease by participating in the sex trade to earn money:

*“This calamity mainly befalls those people who are greedy…” [Educationalist, Female]*

One HIV-uninfected adolescent, while stating that she would be happy to be friends with a peer who is HIV infected, also said that she would not accept a gift from such a person since sharing may lead to some ‘harm’:

*“What you can do if it is a bottle of water you give it to them and tell them you have another bottle. If they give you potatoes [cooked food], just say thanks, better than taking something that may harm you.” [HIV uninfected adolescent, Female, 13 years old]*

Also among the teachers, health care workers and clinicians examples of discrimination, isolation and stigmatization were raised:

*“It is discrimination. You will see a child like this one, he is also part of the family, but you find even when there are family issues to be discussed he is not involved… [Community based health worker, Female]”*

*“……..there is still a lot of stigma” [Hospital bases health worker, Male]*

For HIV infected individuals, perceived stigma was frequently mentioned and in some instances there was even an indication of self –stigmatization:

*“Being isolated by my family members, I reached a point where I felt unwanted in the household…”* (*Mother HIV infected adolescent)*

*“All people know AIDS is a bad disease and even children know that.” (Mother HIV infected adolescent)*

*“It’s like when someone is living with HIV and I don’t have it, then I would feel he has no right to air views or contribute to something because he is not normal.” (Father HIV infected adolescent)*

### Medical adherence: A problem perceived by service providers and caregivers

The HIV infected adolescents themselves did not indicate any issues related to taking antiretrovirals and other prescribed medication. However, several health workers and caregivers indicated that it was challenging to ensure medical adherence for adolescents under their care:

*“When they are younger, their parents usually bring them [to the clinic], but at that age [during adolescence] some of them decline taking drugs. It’s really a challenge. Some of them we’ll have to follow them up to their homes to try to convince them try to give them health education. ……” [Clinical officer, Female]*

*“Recently he mentioned…’Grandma, I am tired of all these medicines, and I do not feel happy’ I told him you will have to continue taking anyway, what else can we do?” [Grandmother, caregiver of an HIV infected adolescent]*

*“The challenge we have is the use of medication, it has been difficult, and I had to chase him a lot, to ensure he takes his medicine…..” [Mother of HIV infected adolescent].*

## Discussion

We investigated the challenges faced by HIV infected adolescents and their caregivers along the Kenyan coast. We observed that the challenges faced by adolescents in rural Kenya could be placed into six major themes: poverty, poor mental and physical health, the lack of a school system that is responsive to their needs, challenges in how to disclose to peers and family members, high levels of stigma in its various forms, and challenges of medical adherence leading to the need for close monitoring. In this setting the burden of poverty seems to exacerbate the negative effects of HIV infection. Based on the discussions with our participants, lack of resources contributed to missed clinic appointments, poor nutrition, and elevated stress for caregivers, further exacerbating the negative impact of HIV infection. Economic empowerment programmes such as the conditional and unconditional cash transfers for orphaned and vulnerable children [14, 15] could be scaled up to enhance the well-being of adolescents living with HIV infection.

Early in the HIV epidemic it was already noted that HIV infection was likely to produce developmental and educational challenges [16]. However, with so few HIV infected adolescents going to school especially in sub-Saharan Africa, little or no attention has been paid to the impact of HIV infection on the educational experience of vertically infected adolescents. There have been studies indicating that both HIV infection and exposure lower educational outcomes [17]. However, fewer studies have actually examined the influence of school on HIV related challenges such as medication. Our study indicates that school related challenges of HIV infected children are becoming prominent and warrant attention. It is a major concern that the adolescents seem to have their educational experiences curtailed following a period of severe illness. There is therefore a need for the educational system within Kenya and other similar settings to think of ways to ensure continued education that is sensitive to medical and developmental needs of children living with chronic conditions.

Previous studies, from both resource rich and resource poor settings, have reported that handling of disclosure remains a salient issue in the lives of HIV infected adolescents [18, 19]. Based on a study in Denmark where a grounded theory analysis that produced a theoretical framework to understand how people approach disclosure, three strategies used to handle disclosure were identified [20]. These strategies are total openness, partial disclosure, and total non-disclosure. In Kilifi, partial disclosure to only a few people within the family context seemed to be the most preferred approach. Similar to the Danish study, the main motivation for using partial disclosure in our setting is to avoid rejection and possible stigmatization. In the Danish study, it was observed that the use of partial disclosure is likely to lead to increased anxiety (over fear of accidental disclosure to or by a third party) and a loss of potential social support. Similar trends were observed in our study; although we did not carry out an in-depth analysis of the issues. Another important issue is the implication of partial disclosure to disease management both at a personal and societal level. To ensure that adolescents can take their medication properly and discuss their HIV status with significant others, there is a need for an intensification of existing efforts and the development of behavioral interventions that equip them with the necessary skills needed to disclose to significant others. This issue becomes even more pertinent as the adolescents move into young adulthood and new ecological contexts and relationships.

Besides disclosing their status, we were also interested in how the adolescents reacted when they were informed that they were HIV infected. Our results indicate that most of them handled the news well showing what can be termed to be a mild reaction to receiving news of the fact that they were living with a chronic condition. These results can be interpreted in two ways. The first is that the efforts that the HIV specialized clinics put into preparing families and counseling them before and after disclosure are succeeding. An alternative explanation may be that adolescents do not fully comprehend the implications of the news related to them being HIV infected. This was also reported in a study in Zimbabwe, where a similar line of inquiry was followed: *“Although most adolescents were able to vividly recall the event when they were first told that they were HIV-positive, many said that they did not fully understand what HIV/AIDS was at the time, or that they were too shocked by the news to grasp any of the HIV/AIDS-related information that was being conveyed”* [21]*.* We cannot make conclusive statements about which of the two potential explanations best explains our observations; future studies are required to understand the complex nature of disclosure in our context. These data are salient in providing service providers sufficient evidence to make recommendations on when, and how to disclose HIV status to vertically infected adolescents.

Stigma and rejection seem to remain prominent in the lives of HIV infected adolescents. The fact that HIV infection remains a stigmatized disease three decades later remains an issue of concern both for those living with HIV infection and for policy makers [22], since it negatively impacts on the outcomes of HIV infected individuals. As suggested in the literature [23, 24], the fear of stigma influences disclosure, disease management, and social relations. There is an urgent need to get a greater understanding of stigma and its ramifications and to develop intervention programmes aimed at combating HIV-related stigma especially in adolescents and young adults.

As children grow older and start taking more responsibility for their medication, concerns regarding clinical and treatment adherence have been expressed across many contexts globally. Similar trends were observed in our study where caregivers and health care providers indicated that this was one of their major worries. A recent study in Kilifi indicated that the rates of ARV treatment failure and development of resistance to the drug regime were much higher among adolescents and young adults [25], which may be an indication that they are not taking their drugs as expected. In another study in Zimbabwe, it was also observed that adolescents and young adults were at 4 times higher risk of treatment failure compared to young children, which led the authors to recommend the provision of *“adapted adherence support and service delivery models for both adolescents and young adults”* [26]*.* Our study found that barriers to adherence to medication included stigma, lack of disclosure, and peer influence. These barriers to medical adherence are largely behavioral; suggesting an urgent need for behavioral interventions to enhance rates of medical adherence among HIV infected adolescents. Furthermore, these results provide further support for the development of long acting ARVs to facilitate medical adherence.

### Implications for intervention and care

**Fig. 2:**
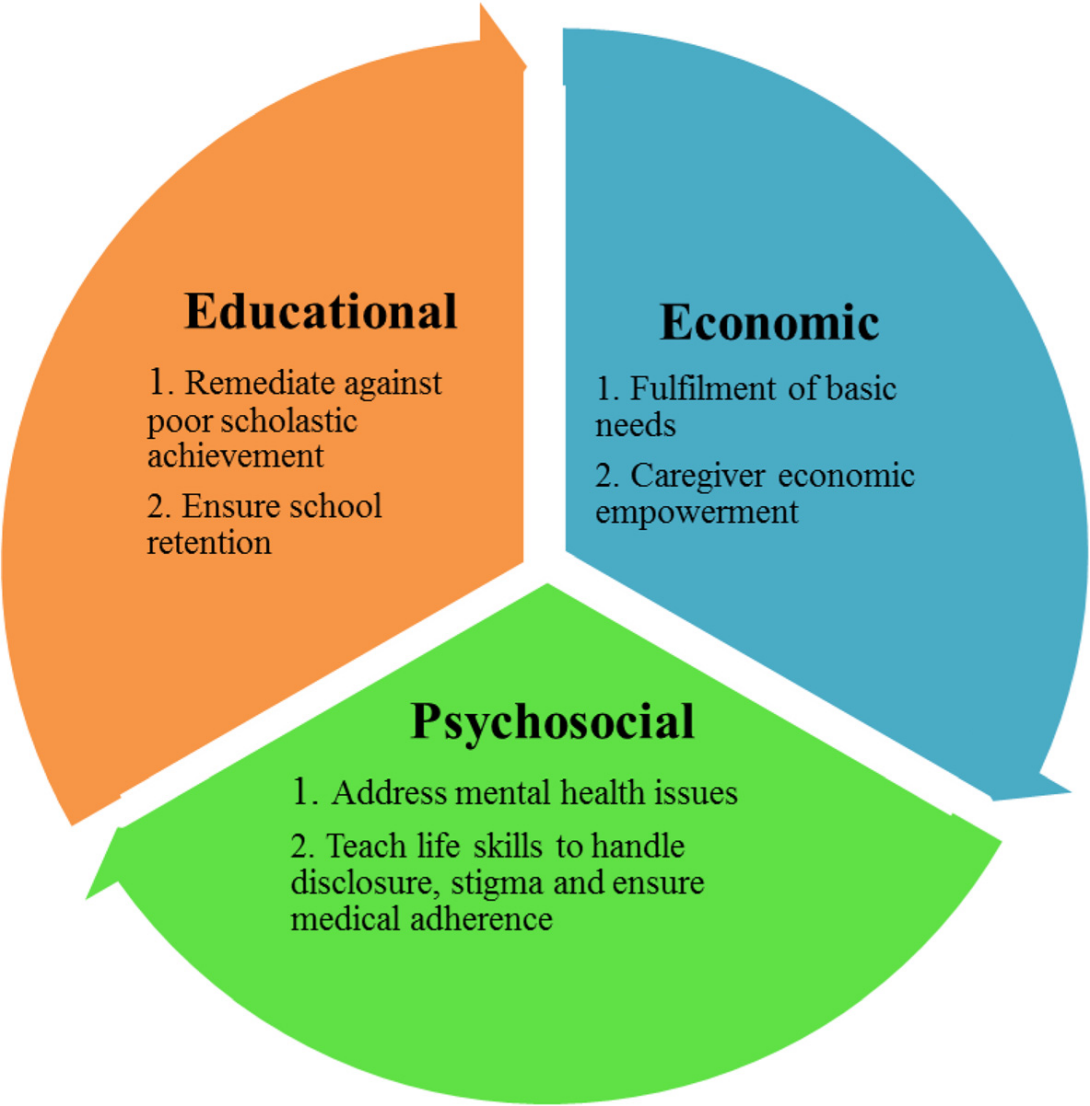
Potential areas of interventions according to our data

Our results have various implications for the kind of care that needs to be provided to HIV infected adolescents within Kilifi and other similar settings. The most important implication is that if these adolescents are to thrive there is need to provide a comprehensive and multi-sectorial programme that addresses their various challenges, many of which are not medical (see Fig. 2). Our results indicate that we need educational programs targeted at rehabilitating those who fall behind, economic empowerment to address poverty related changes and psychosocial interventions which assist the adolescents deal with mental health problems and teach them the required life skills.

### Limitations

First, the sample size is relatively small and we use a single qualitative approach. A multimethod approach would have allowed for the triangulation of the information received. Second, being a cross-sectional study we are only able to present a snapshot of perceived challenges, richer information would have been captured had we used a longitudinal approach. Lastly, due to ethical challenges associated with speaking to adolescents who have not fully been disclosed to their HIV status, we only recruited adolescents who were fully aware of their status and were attending the clinics. This may bias the results and may not make them generalizable to those who are either not fully disclosed or those who are not attending specialized HIV clinics regularly.

## Conclusions

Our results highlight the fact that vertically acquired HIV infected adolescents face a host of challenges likely to exacerbate the negative effects of HIV infection. We therefore conclude that, for vertically infected adolescents to thrive and achieve their full potential there is a need to develop and implement a multisectorial evidence-based intervention program aimed at addressing the full spectrum of their challenges.

## Additional file

Additional file 1:
**Interview Guide.** (DOC 41 kb)
