# Supplementary material for: ‘Everyone has a secret they keep close to their hearts’: challenges faced by adolescents living with HIV infection at the Kenyan coast
Source: BMC Public Health. 2016 Feb 29;16:197. doi: 10.1186/s12889-016-2854-y (PMC4772469; doi:10.1186/s12889-016-2854-y)
Supplement: Additional file 1: — Interview Guide. (DOC 41 kb) [file 12889_2016_2854_MOESM1_ESM.doc]

**Appendix 1: Interview Guide**

**Adolescents living with HIV [[1]](#endnote-2), [[2]](#endnote-3), [[3]](#endnote-4)**

I would like us to discuss with you about living with HIV as an adolescents**.**

- Can you explain to me how you came to know that you are living with HIV? Let us discuss your feelings, thoughts and actions when you learnt about it.
- In your opinion, how has living with HIV affected/ impacted on your life?
- What are the challenges you face living with HIV?
- Do you think living with HIV has affected your relationship with other people? (Ask for the participants to provide specific examples when talking about this).
- Whom have you told about your HIV status? (Why- Haven’t you told the others?)
- I would like us to talk about your educational experience. How are you doing in school? What challenges do you face?

**Caregivers of Adolescents living with HIV**

- Have you discussed with your child/ or the child you are caring for about their HIV status? How did they react when they were fully disclosed to that they were HIV positive?
- In your opinion, how has living with HIV affected/ impacted on their lives?
- What are the challenges they face living with HIV?
- Whom have you told about the child’s HIV status? (Why- Haven’t you told the others?)
- I would like us to talk about their educational experience. How are they doing in school? What challenges do they face at school?

**Interview Guide for use with health care providers (doctors, nurses counsellors and community health workers)**

- In your opinion/experience, how does HIV impact on the lives of adolescents [age 12-17]
- What are the challenges faced by adolescents living with HIV?

**Interview Guide for use with Teachers/educationalists/school matrons/adolescents living without HIV**

- In your opinion/experience, how does HIV affect/impact on the lives of adolescents [age 12-17]
- What are the challenges faced by adolescents living with HIV? Any specific challenges faced in the educational setting or at school?

1. These questions are a sub-sample of the interview and represent only the questions relevant to the paper presented here. Additionally, probes are not presented as they were largely presented based on the participants response. [↑](#endnote-ref-2)
2. These are a direct translation from Kiswahili; the language that was largely used during the interview [↑](#endnote-ref-3)
3. Questions that were presented as preambles to settle in the participants are not presented. [↑](#endnote-ref-4)
